# A specific SNP-based multiplex PCR assay for the simultaneous identification of two biological ingredients for the Chinese patent medicine, Danggui Buxue pill

**DOI:** 10.3389/fphar.2023.1098598

**Published:** 2023-05-11

**Authors:** Yinrong Liu, Yingying Yang, Zishan Zhou, Jia’er Fan, Jianxin Diao, Zhi Chao, Enwei Tian

**Affiliations:** ^1^ School of Traditional Chinese Medicine, Southern Medical University, Guangzhou, China; ^2^ Shenzhen Hospital of Guangzhou University of Chinese Medicine (Futian), Shenzhen, China; ^3^ Guangdong Provincial Key Laboratory of Chinese Medicine Pharmaceutics, Guangzhou, China; ^4^ Guangdong Provincial Engineering Laboratory of Chinese Medicine Preparation Technology, Guangzhou, China

**Keywords:** multiplex PCR assay, Chinese patent medicine, molecular identification, nrITS, Angelicae Sinensis Radix, Astragali Radix

## Abstract

**Background:** An increasing number of Chinese patent medicines (CPM) have been widely used in East Asian and North American countries, and the safety and efficacy of CPM have highly attracted public attention. However, it is difficult to supervise the authenticity of multiple biological ingredients within CPM based on microscopic inspection and physical and chemical detection. The raw materials may have similar characteristics of tissue structures and ergastic substances or similar chemical composition and contents when substitutes and/or adulterants are added. DNA molecular markers have been used to distinguish the biological ingredients within CPM based on conventional PCR assay. However, it was proved to be time- and labor-consuming and reagent-wasting, as multiple PCR amplification strategies were required for identifying the complex species composition within CPM. Here, we took the CPM (Danggui Buxue pill) as an example and aimed to establish a specific SNP-based multiplex PCR assay and simultaneously determine the authenticity of the two biological ingredients (Angelicae Sinensis Radix and Astragali Radix) within this CPM.

**Methods:** We, respectively, designed the species-specific primers based on highly variable nrITS for discriminating Angelicae Sinensis Radix and Astragali Radix from their common substitutes and adulterants. The specificity of the primers was checked through conventional PCR assay and multiplex PCR assay. Furthermore, we used a handcrafted Danggui Buxue pill sample (DGBXP) to optimize annealing temperatures for the primers with multiplex PCR, and the sensitivity was also assessed. Finally, fourteen batches of commercial Danggui Buxue pills were used to verify the stability and practicability of the established multiplex PCR assay.

**Results:** Two pairs of highly species-specific primers for amplifying Angelicae Sinensis Radix and Astragali Radix were screened, and our established multiplex PCR assay showed high specificity and sensitivity (lowest detection concentration: 4.0 × 10^−3^ ng/μL) at an optimal annealing temperature of 65°C. The method could simultaneously identify both biological ingredients within the Danggui Buxue pill.

**Conclusion:** The specific SNP-based multiplex PCR provided a simple, time-, and labor-saving method for the simultaneous identification of the two biological ingredients within Danggui Buxue pills. This study was expected to provide a novel qualitative quality control strategy for CPM.

## 1 Introduction

Chinese patent medicines (CPMs) have definite curative effects in treatment, with insignificant toxicity and side effects and are convenient in clinical use (Zhuang et al., 2020; Ye and Zhang., 2021) and are gradually widely used in East Asian, European, and North American countries (Zhang, 2017). Despite the increasing demand and consumption, concerns about the quality and safety of CPMs remain because it still has some limitations, especially in the authenticity of adding biological ingredients within CPM. Substitute and/or adulterant addition will seriously affect clinical safety and effectiveness due to differences in clinical efficacy among different crude drugs. However, the complex prescriptions (multiple biological ingredients) and diverse chemical components make it difficult to accurately identify the origin of raw botanical drugs within CPM (Yang et al., 2020).

Over the past decades, physical and chemical methods such as microscopic inspection, thin-layer chromatography (TLC), high-performance liquid chromatography (HPLC), and gas chromatography (GC), have widely been used to qualitatively and quantitatively analyze tissue structures, ergastic substances, or indicative components within CPMs for quality control (Sun et al., 2009; Gao and Niu, 2020; Li et al., 2020). However, there still exist some challenges pertaining to accuracy or specificity because these methods are often affected by environmental and subjective factors, or quantitative variations of characteristic components within CPMs impede the determination of their biological components (Hon et al., 2003; Hui et al., 2004; Zhao and Zhao, 2014; Fu, 2019). DNA molecular markers and DNA barcoding technologies based on the variation characteristics of gene fragments have been widely used in species identification during the past years (Raclariu-Manolica et al., 2021). These technologies are less affected by environmental factors, sample forms, and chemical composition, or the experiences of operators and thus show significant advantages in the authentication of traditional Chinese medicine (TCM) and CPM (Yang F. et al., 2018). However, many times, PCR reactions are required to distinguish the complex biological ingredients within CPM using a conventional PCR assay, which is not only time- and labor-consuming but also reagent-wasting. Recently, DNA metabarcoding and shotgun metagenomics technologies were the mostly used detection methods for mixed biological samples within CPM (Xin et al., 2018; Liu et al., 2021). Although multi-biological ingredients could be identified, the PCR amplification efficiency of universal DNA barcode primers was affected by severe DNA degradation of CPM when using DNA metabarcoding, and both methods were relatively more expensive. Furthermore, the related bioinformatics analysis also presented significant challenges, such as false positives caused by read mapping in conserved regions and difficulties in the assembly of high-similarity sequences or low-variability sequences (Xin et al., 2018). On the basis of traditional PCR, recently, the multiplex PCR method has been developed, which might add more than two primers in one reaction system and could simultaneously amplify multiple targeted genes in one reaction (Dong et al., 2020). Zhang et al. (2022) successfully detected 10 kinds of foodborne pathogens using the multiplex PCR method. Thus, it is much faster and more economical than conventional PCR (Chen M. J. et al., 2005; Dong et al., 2020), and it also does not need to perform complicated bioinformatics analysis. Multiplex PCR is used to determine whether the genuine crude drugs were doped with adulterants (Wang et al., 2012; Jiang et al., 2017), yet not applied in the detection of CPM containing multiple biological ingredients. Herein, we aimed to establish a multiplex PCR assay that could simultaneously identify all authentic biological ingredients within CPM.

The Danggui Buxue pill is composed of two crude drugs, Angelicae Sinensis Radix (“Danggui” in Chinese) and Astragali Radix (“Huangqi” in Chinese), which have the therapeutic functions of nourishing Qi and blood. It has been frequently used to treat Qi and blood deficiency and weakness in gynecology (Hu et al., 2020). The two biological components, Angelicae Sinensis Radix and Astragali Radix, found in Danggui Buxue pills were originated from the dry roots of *Angelica Sinensis* (Oliv.) Diels and *Astragalus membranaceus* (Fisch.) Bge. var. *mongholicus* (Bge.) Hsiao [or *Astragalus membranaceus* (Fisch.)], respectively (Chinese Pharmacopoeia Commission, 2020). As the two crude drugs are both precious Daodi drugs in China, there are many adulterants instead of genuine ones added in CPM, driven by the economic benefits (Wang et al., 2016). Previous studies have reported that the roots of *Levisticum officinale* Koch.*, Angelica decursiva* (Miquel) Franch. & Sav., and *Angelica dahurica* (Hoffm.) Benth. & Hook. f. ex Franch. & Sav. were genuine “Danggui” (Chen, 2010; Wang et al., 2016), while substitutes and counterfeit products of “Huangqi” usually were found in the roots of *Phyllolobium chinense* Fisch., *Medicago sativa* L., *Alcea rosea* L., and *Caragana sinica* (Buc’hoz) Rehder (Wang et al., 2013; Zhang et al., 2018). These biological ingredients were intentionally or unintentionally misused in Danggui Buxue pills, which might seriously affect the clinical effectiveness of this CPM and even cause medication safety issues.

In this study, we selected the highly variable nrITS (internal transcribed spacer rRNA gene) as the targeted DNA barcode, which has been widely recognized for its usefulness in plant species identification (Chen et al., 2010; Parvathy et al., 2015) and designed specific SNP primers for discriminating Angelicae Sinensis Radix and Astragali Radix from their common adulterants. Furthermore, we tried to establish a rapid and accurate multiplex PCR assay for the simultaneous determination of Angelicae Sinensis Radix and Astragali Radix within the Danggui Buxue pill. Hopefully, this method could determine the authenticity of authentic biological ingredients in Danggui Buxue pills and also provide a methodological reference and supplement for qualitative quality control of the CPM.

## 2 Materials and methods

### 2.1 Sample collection and preparation

In total, 35 specimens, including the original plants of genuine Angelicae Sinensis Radix, Astragali Radix, and their common adulterants from different production areas in China, were collected (sampling information is listed in Table 1). The specimens were identified by Associate Professor Enwei Tian and Professor Zhi Chao from the School of Traditional Chinese Medicine, Southern Medical University (SMU), and were deposited in the herbarium of the School of TCM, SMU. The fresh leaves were preserved in gel-dried silica for genomic DNA extraction and further used to evaluate the specificity of the designed primers. To optimize the reaction conditions of the multiplex PCR method, the Danggui Buxue pill was handcrafted according to the guidelines of the Drug standard of the Ministry of Health of the People’s Republic of China—traditional Chinese medicine formulation (Pharmacopoeia Committee of the People’s Republic of China, 1995) and named DGBXP. The detailed preparation procedure is as follows: two different doses (160 g of Angelicae Sinensis Radix and 400 g of Astragali Radix) were crushed into fine powder, which was further sieved and mixed. Then, 30 g–40 g of refined honey was added, an appropriate amount of water was added to the powder, and the pills were panned. Afterward, the pills were dried. Four batches of adulterated Danggui Buxue pills were handcrafted referring to the aforementioned preparation (Batch 1: genuine Angelicae Sinensis Radix and Astragali Radix plus all adulterants; Batch 2: genuine Angelicae Sinensis Radix plus adulterants of Astragali Radix and Angelicae Sinensis Radix; Batch 3: genuine Astragali Radix plus adulterants of Angelicae Sinensis Radix and Astragali Radix; Batch 4: all adulterants), which were used to further test the specificity of the designed primes.

**TABLE 1:** Sampling information of the original plant of Angelicae Sinensis Radix, Astragali Radix, and their adulterants.

Species	Voucher no.	Locality	Coordinate
*Angelica sinensis* (Oliv.) Diels	MX1	Dingxi, China	N38° 34′ 39″ and E106° 13′ 32″
	MX2		
	MX3		
	GS1		
	GS2		
	GS3		
	YN1	Heqing, China	—
	YN2		
	YN3		
	YN4		
*Levisticum officinale* Koch	ODG1	Nanjing, China	N32° 03′ 38″ and E118° 50′ 20″
	ODG2		
	ODG3		
*Angelica decursiva* (Miquel) Franch. and Sav	ZN1	Shaoguan, China	N24° 54′ 50″ and E113° 57′ 50″
	ZN2		
	ZN3		
*Angelica dahurica* (Hoffm.) Benth. and Hook. f. ex Franch. and Sav	AG1	Baoding, China	N38° 25′ 11″ and E115° 19′ 37″
	AG2		
	AG3		
*Astragalus membranaceus* (Fisch.) Bge. var. *mongholicus* (Bge.) Hsiao	ZY1	Dingxi, China	N34° 57′ 35″ and E104° 32′ 08″
	ZY2		
	DT1	Chengde, China	N40° 40′ 12″ and E117° 40′ 12″
	DT2		
	GM1	Lanzhou, China	N36° 48′ 30″ and E103° 10′ 50″
	GM2		
*Phyllolobium chinense* Fisch.	SWZ1	Weinan, China	N34° 49′ 46″ and E110° 04′ 17″
	SWZ2		
*Medicago sativa* L	ZMX1	Beijing, China	N40° 01′ 52″ and E116° 17′ 34″
	ZMX2		
	ZMX3		
*Alcea rosea* L	SK1	Shanghai, China	N31° 05′ 02″ and E121° 11′ 24″
	SK2		
	SK3		
*Caragana sinica* (Buc’hoz) Rehder	JJE1	Shanghai, China	N31° 05′ 02″ and E121° 11′ 24″
	JJE2		

To determine the stability and practicability of the multiplex PCR assay, 14 batches of commercial Danggui Buxue pills were purchased from two manufacturers in China and numbered BX1–BX8 (Jilin Zixin Pharmaceutical Industrial Co., Ltd., China; batch nos: 190204, 190302, 190306, 190308, 190311, 190809, and 190506) and BX9–BX14 (Shangqiu Jinma Pharmaceutical Co., Ltd., China; batch nos: 20092021, 21062221, 19123021, 21032722, 21041122, and 20112321).

### 2.2 DNA extraction

The modified CTAB method (4% CTAB for cell lysis and polysaccharide removal and 2 volumes of ice-cold 70% methanol for DNA precipitation) was used to extract the total genomic DNA of the dried leaves from the 35 samples (Murray and Thompson, 1980; He et al., 2011). For the 14 batch samples of commercial Danggui Buxue pills, handcrafted samples (DGBXP), and four batches of handcrafted and adulterated Danggui Buxue pills (B1∼ B4), the aforementioned modified CTAB method combined with the silica-based spin column DNA purification kit (Cat.#DP204-03 and Lot#X0902; Tiangen, Bejing, China) was used for genomic DNA extraction. Considering the potential issues of the DNA degradation of commercial and handcrafted samples, we increased the dosage of samples (2 g powder) for DNA extraction, which was much more than that (50 mg–100 mg) of dried leaves using the classic CTAB method (Murray and Thompson, 1980). The concentration and integrity of genomic DNA were assessed using a NanoDrop 1000 UV/Vis spectrophotometer (Thermo Scientific, Wilmington, DE, USA) and 1.5% agarose gel electrophoresis, respectively.

### 2.3 Design of species-specific primers based on nrITS sequences

Fourteen and sixteen nrITS sequences for the original plants of Angelicae Sinensis Radix, Astragali Radix, and their adulterants were downloaded from GenBank (NCBI: https://www.ncbi.nlm.nih.gov/) (Supplementary Table S1, Supplementary Table S2). Quality control for these sequences complied with the five simple quality control guidelines for establishing basic authenticity and reliability (Nilsson et al., 2012). Afterward, the sequences were aligned using MEGA v7.0.14 software (Kumar et al., 2016) and used to search for highly variable SNP sites. Primer Premier 5 software (https://www.premierbiosoft.com/primerdesign/index.html) was used to design primers for the specific ITS regions of *A. sinensis*, *A. membranaceus* var. *mongholicus,* and *A. membranaceus*. The designed primers were evaluated according to the standards of Li et al. (2015) and Sathirapongsasuti et al. (2020). The primers were synthesized by Sangon Biotech Co., Ltd. (Shanghai, China). After the pre-experiment, two species-specific primer pairs (DG and HQ) were tentatively selected for identifying Angelicae Sinensis Radix and Astragali Radix (Table 2). The conservation of the specific SNPs for Angelicae Sinensis Radix and Astragali Radix had been determined by phylogenetic trees (neighbor-joining tree), which were generated by the downloaded ITS sequences, respectively (Supplementary Figure S1, Supplementary Figure S2).

**TABLE 2:** Sequence information of the two pairs of species-specific primers selected for the identification of Angelicae Sinensis Radix and Astragali Radix.

Primer	Primer sequence (5′ → 3′)	*T*_a_	Product size (bp)
DG	F: GGC​TTT​GGT​CCC​TTG​TAT​G	50°C–67.6°C	349
	R: CAC​GAG​GAG​TGA​GTG​GTT​G		
HQ	F: GCA​CCA​CGA​CCT​CCC​TTT​G	50°C–67.6°C	381
	R: GCCATCATTCGCCCTAAA		

### 2.4 Validating the specificity of the two primers pairs based on conventional PCR and multiplex PCR

The two selected primers were used in conventional PCR amplification of genuine Angelicae Sinensis Radix (six samples: MX1∼MX3 and GS1∼GS3) and its adulterants (nine samples: ODG1, ODG2, ODG3, ZN1, ZN2, ZN3, AG1, AG2, and AG3) and Astragali Radix (six samples: GM1, GM2, DT1, DT2, ZY1, and ZY2) and its adulterants (JJE1, JJE2, ZMX1, ZMX2, ZMX3, SWZ1, SWZ2, SK1, SK2, and SK3), respectively (Table 1). Four batches (B1∼B4) of adulterated Danggui Buxue pills were used to further test the specificity of the two selected primers using multiplex PCR. The 20 uL PCR reaction system contained 2 uL of the DNA template, 1.6 μL of 10 × buffer (Mg^2+^ free), 2.5 uM of MgCl_2_, 300 uM of dNTP mixture, 2U of rTaq DNA polymerase (Takara, Dalian, China) and 0.5 uM of HQ primers, or/and 0.25 uM of DG primers. PCR amplification was performed with the following procedure: one pre-denaturation cycle of 5 min at 94°C, 35 cycles of 94°C for 50 s, 65°C for 30 s, 72°C for 90 s, and followed a final extension at 72°C for 7 min. PCR products were analyzed on 1.2% agarose gel and visualized by the electrophoresis gel imaging system (Peiqing, Shanghai, China).

### 2.5 Reaction condition optimization for multiplex PCR assay

#### 2.5.1 Annealing temperature optimization

The genomic DNA extracted from the handcrafted sample (DGBXP) was selected as the DNA template, which was used for multiplex PCR amplification for identifying Angelicae Sinensis Radix and Astragali Radix with their respective species-specific primers*.* The annealing temperatures ranging from 50 °C to 72°C (50°C, 51°C, 52.4°C, 54.6°C, 57.2°C, 59.7°C, 62°C, 65.1°C, 67.6°C, 69.6°C, 71.1°C, and 72°C) were set to determine the optimal temperatures (range) for the multiplex PCR assay.

#### 2.5.2 The sensitivity detection of the multiplex PCR assay

To detect the sensitivity of the multiplex PCR method, 10-fold dilutions of genomic DNA templates extracted from the handcrafted sample (DGBXP) were used for multiplex PCR, which started at the genomic DNA concentrations of 40 ng/μL to 4.0 × 10^–4^ ng/μL (six concentration gradients). Multiplex PCR products were visualized by 1.5% electrophoresis gel, which could determine the lowest detection concentration of the multiplex PCR assay.

#### 2.5.3 Authenticity identification of the two biological ingredients within commercial Danggui Buxue pills

To determine the stability and practicability of the multiplex PCR assay, fourteen batch samples (BX1∼BX14) of commercial Danggui Buxue pills were used for the identification of the two biological ingredients. According to the optimal reaction conditions, the 14 genomic DNA samples were used as the templates, and each DNA template was simultaneously amplified with the two confirmed species-specific primers in one reaction for determining the two biological ingredients within Danggui Buxue pills.

## 3 Results

### 3.1 The quality of genomic DNA extracted from plant materials, handcrafted and commercial Danggui Buxue pills

The genomic DNA extracted from plant materials performed well on a single DNA band in agarose gel, but not for handcrafted and commercial Danggui Buxue pill samples, which suggested that serious DNA degradation occurred within these samples due to the processing of raw materials and the addition of various excipients. The concentration detection results showed that they varied from 60 to 200 ng/μL and from 10 to 400 ng/μL for plant materials and Danggui Buxue pill samples (including handcrafted samples), respectively. The A260/A280 information and the DNA electrophoretogram of handcrafted DGBXP, of the 14 samples of commercial Danggui Buxue pills, and of the four samples of handcrafted Danggui Buxue pills (adulterated) are shown in Supplementary Table S3 and Supplementary Figure S3.

### 3.2 Determination of the specificity of the two specific SNP primers

**FIGURE 1:**
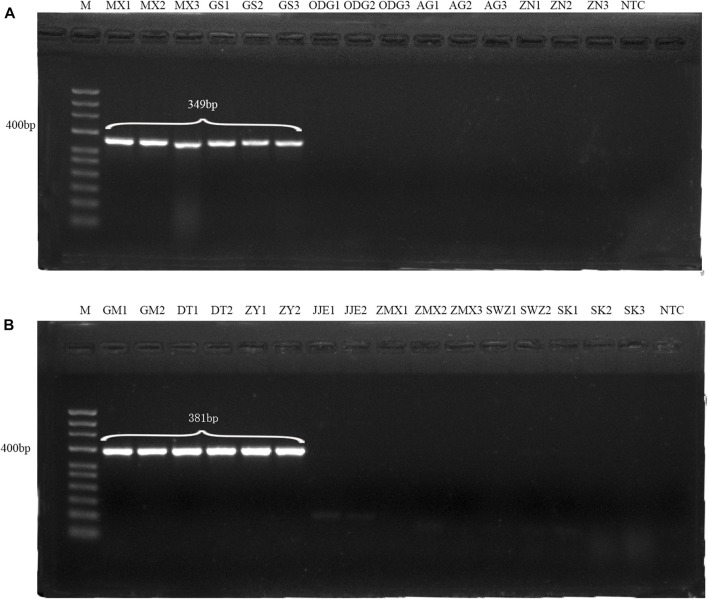
Validation of specific identification of Angelica Sinensis Radix with DG primers **(A)** and Astragali Radix with HQ primers **(B)**. All samples were labeled with their names above the lanes(MX1, MX2, MX3, GS1, GS2 and GS3 indicate the genuine Angelica Sinensis Radix, and GM1, GM2, DT1, DT2, ZY1 and ZY2 indicate the genuine Astragali Radix; the others indicate adulterants). M: 50 bp ladder DNA marker; NTC: negative control.

Conventional PCR amplification with the single pair of primers (DG and HQ) generated DNA bands of 349 bp and 381 bp for Angelicae Sinensis Radix and Astragali Radix, respectively, and no amplification signals were observed for the adulterant samples (Figures 1A,B; Supplementary Figure S4). It demonstrated that DG and HQ primers had strong specificity and could be used for identifying genuine Angelicae Sinensis Radix and Astragali Radix. The consistent results were also presented on the specific amplification of four batches (B1∼B4) of adulterated Danggui Buxue pills (Supplementary Figure S5).

### 3.3 Optimization of annealing temperatures for multiplex PCR assay

**FIGURE 2:**
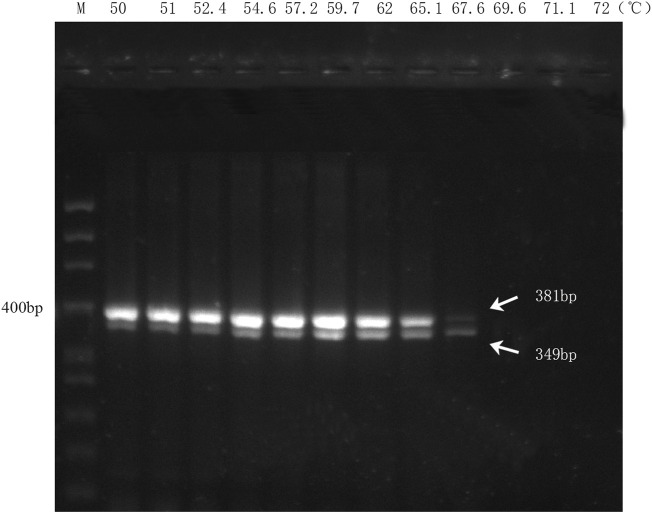
Annealing temperature screening of the multiplex PCR with DG and HQ primers. M: 50 bp ladder DNA marker. Lanes 1 to 12: The gradients of annealing temperature ranged from 50°C to 72°C.

The results of the multiplex PCR amplification showed that the two biological ingredients within the handcrafted sample (DGBXP) could be successfully amplified at the temperature range of 50°C–67.6°C (Figure 2). Based on the amplification signals, the optimal temperature range of the multiplex PCR was 54.6°C–65.1°C for both ingredients. To ensure the specificity of the primers, we chose 65°C as the optimal temperature to detect the sensitivity of the multiplex PCR, which was also further applied for the identification of the biological ingredients within commercial Danggui Buxue pills.

### 3.4 Assessment of the sensitivity of the multiplex PCR assay

**FIGURE 3:**
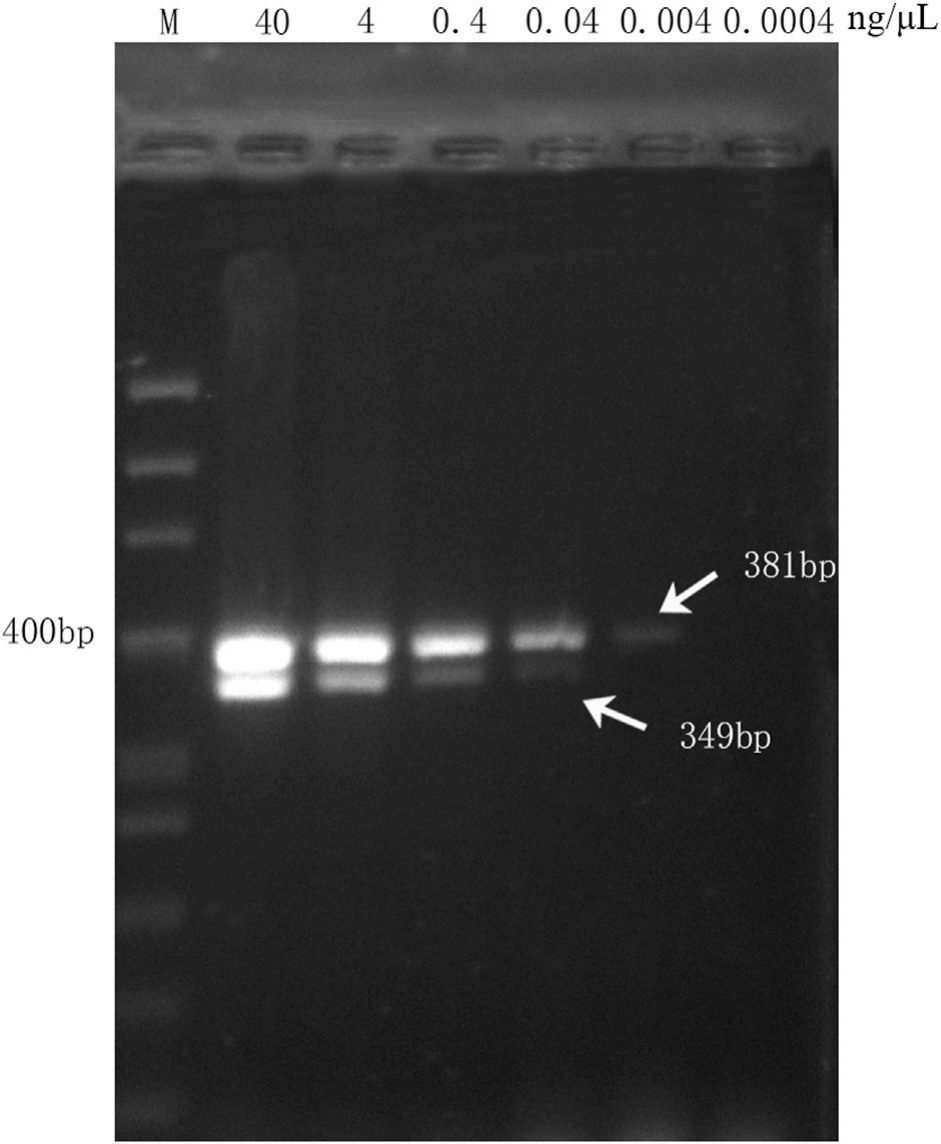
Sensitivity analysis of the multiplex PCR assay with different concentration gradients of genomic DNA derived from handcrafted samples (DGBXP). M: 50 bp ladder DNA marker; Lane 2 ∼ Lane 7: The concentration gradients of DGBXP genomic DNA ranged from 40 ng/μL to 0.0004 ng/μL.

To assess the sensitivity of the multiplex PCR assay, six DNA concentration gradients of DGBXP samples were set, which started at a concentration of 40 ng/μL (initial concentration) and were successively diluted 10 times until the terminal concentration of 4.0 × 10^−4^ ng/μL. The multiplex PCR assay was capable of amplifying both targeted fragments (349 bp and 381 bp) when the genomic DNA of the DGBXP sample was diluted from 40.0 to 4.0 *×* 10^*–*2^ ng/μL. Then, the genomic DNA was further diluted at a concentration of 4.0 *×* 10^−3^ ng/μL, and the targeted gene fragment (HQ, 381 bp) for Astragali Radix was still successfully amplified, but with a weak signal. However, no amplification signal was present at the 349 bp position (DG) for Angelicae Sinensis Radix (Figure 3). The results suggested the lowest detection concentration was 4.0 *×* 10^*–*2^ ng/μL and 4.0 *×* 10^*–*3^ ng/μL for Angelicae Sinensis Radix and Astmgali Radix, respectively.

### 3.5 Authentication of the two biological ingredients within commercial Danggui Buxue pills

**FIGURE 4:**
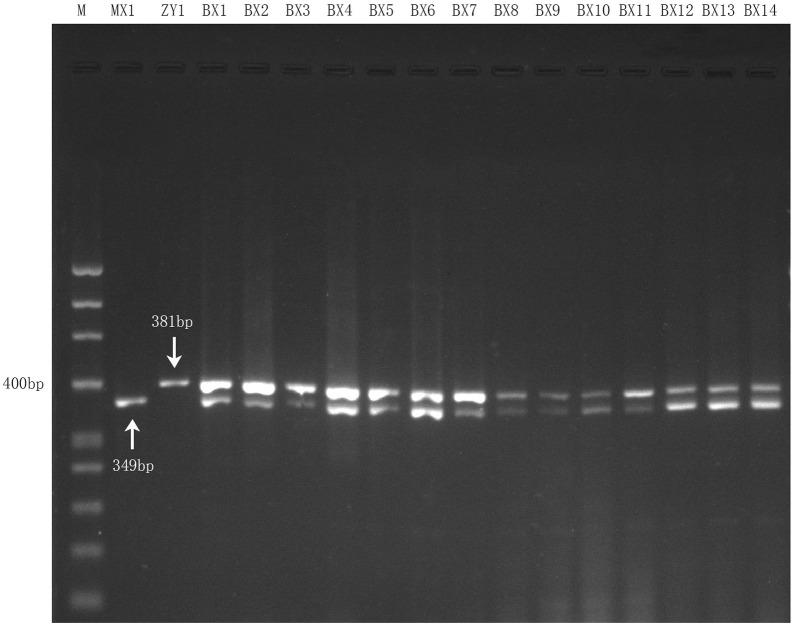
Multiplex PCR amplification results for 14 commercial Danggui Buxue pill samples with the two species-specific primers. Lane 1 and Lane 2 indicated the two reference crude drugs, MX1 (Angelicae Sinensis Radix) and ZY1 (Astragali Radix), respectively. Lane 3 to Lane 16: the 14 commercial samples.

Fourteen genomic DNA samples of commercial Danggui Buxue pills were respectively amplified with both species-specific primers (DG and HQ) in one reaction according to the optimal reaction conditions of the multiplex PCR assay. The results showed that all the commercial samples could be simultaneously amplified and showed two bands (349 bp and 381 bp) (Figure 4), which suggested that the multiplex PCR assay was stable, accurate, and suitable for detecting Angelicae Sinensis Radix and Astragali Radix within commercial Danggui Buxue pills.

## 4 Discussion

### 4.1 Challenges for DNA extraction of CPM

The quality of genomic DNA is the key to the molecular identification of the biological ingredients within CPM (Jing et al., 2021). However, during the preparation, the raw medical materials are always processed by all kinds of processing methods, such as decocting, calcining, stir-frying, and fermenting (Chen, 2018), which may result in severe DNA degradation and affect DNA quality and quantity (Karni et al., 2013). Meanwhile, the addition of a variety of pharmaceutical excipients, such as honey, starch, and sugar, also causes significant difficulties in DNA extraction (Sajali et al., 2018). Therefore, the methods formulated for genomic DNA extraction depend on the different dosages and specific preparation processes of CPM. In this study, we used the modified CTAB method, which has been widely proven to be suitable for DNA extraction of plant-derived materials (He et al., 2011). Approximately 4% CTAB (the concentration of NaCl was increased to 2.5 M) was used for complete cell lysis and the removal of polysaccharides (Cui et al., 2006), and 2 volumes of ice-cold 70% methanol were added for DNA precipitation overnight, which allowed obtaining high quality of genomic DNA. In order to extract sufficient genomic DNA for further study, we increased the dosage of commercial Danggui Buxue pill samples. After comparing different dosages of samples (0.1 g, 0.5 g, 1 g, and 2 g), we determined the sample dosage of 2 g for DNA extraction, which has been proved to be sufficient for subsequent PCR amplification. Many previous studies also suggested that more than 100 mg should be used for DNA extraction (Gou et al., 2018). DNA purity is another very important factor that affects PCR amplification. Some impurities, such as polysaccharides, polyphenols, and proteins mixed within genomic DNA, will also inhibit PCR amplification (Hedman et al., 2013; Sajali et al., 2018). In order to obtain high-purity genomic DNA, we carried out the CTAB method combined with the silica-based spin column DNA purification kit to extract genomic DNA from commercial samples, and our validation results of PCR amplification with species-specific primers suggested that the method was effective.

### 4.2 Selection of DNA barcodes and molecular markers and the designing principles for specific primers

To select an appropriate DNA barcode for designing species-specific primers, we aligned the sequences of four universal barcodes (ITS, *matK*, *rbc*L, and *trn*H-*psb*A) among the original plant species of Angelicae Sinensis Radix, Astragali Radix, and adulterants and further checked their variation information. Consistent with previous studies (Parvathy et al., 2015), ITS featured much more variables and informative sites, which had already been recognized as a perfect molecular marker in the identification of angiosperms. On the other hand, SNP genotyping with allele-specific PCR is a powerful tool for the authentication of botanical drugs (Wang et al., 2012). In this study, we focused on the variable targeted gene ITS to design allele-specific primers. The reasonable product size of the SNP-based allele-PCR amplification to balance the amplification success rate and resolution of the targeted gene fragments is worth mentioning. Generally, the smaller targeted gene fragment usually shows a higher amplification success rate for the highly processed raw medicinal materials, which makes it much more difficult for designing appropriate primers because of insufficient variation sites harbored in limited gene fragments (Yang J. F. et al., 2018). In this study, the product sizes of amplified gene fragments were less than 400 bp (349 bp and 381 bp, respectively) in length, and these results suggested that both pairs of primers had excellent amplification success rates. Chen Y. et al. (2005) found that although mechanical grinding and high-heat processes would cause DNA degradation, they still amplify approximately 400 bp of DNA fragments, which is consistent with the results of PCR amplification in this study. However, for some other CPMs requiring stricter processing, it is recommended that the size of the amplified product should be around 100–250 bp in length (Yang F. et al., 2018; Gao et al., 2019). Moreover, the difference between product sizes of multiplex PCR amplification with multiple primers is also a major concern. Liu et al. (2018) recommended that the differences in multiple PCR product sizes visualized by gel electrophoresis should not be less than 50 bp, and Zhou et al. (2005) used capillary electrophoresis to distinguish the difference (17 bp) of multiple PCR products. In fact, in this study, the product size difference of approximately 30 bp could still be distinguished by gel electrophoresis. To ensure the specific binding of primers with targeted DNA fragments, we suggest keeping the difference sizes of different targeted gene amplification products as large as possible, which will be conducive to the separation of different targeted fragments in gel electrophoresis. In summary, the product size differences of multiplex PCR for different biological ingredients depend on the number of primers, the difficulties of primer designing, the degree of degradation of the DNA template, and the detecting method.

### 4.3 Assessment of the practicability of the multiplex PCR method in commercial CPM

Detection of Angelicae Sinensis Radix and Astragali Radix within 14 batches of commercial Danggui Buxue pills using multiplex PCR assay yielded positive results for both ingredients. In order to further verify the accuracy of the multiplex PCR assay, the PCR products were sequenced and BLASTed in NCBI (https://blast.ncbi.nlm.nih.gov/Blast.cgi). The similarity compared with ITS sequences of *Angelica sinensis* and *Astragalus mongholicus* in GenBank was 100% (sequence coverage: 100%). Our results indicated that the multiplex PCR assay was highly specific and could simultaneously authenticate Angelicae Sinensis Radix and Astragali Radix with the two pairs of primers. Although all the genuine crude drugs were added in the 14 batches of commercial samples, the sampling errors should not be ignored, and the supervision department should still strengthen the monitoring, as many adulterants of Angelicae Sinensis Radix and Astragali Radix have been reported (Chen, 2010; Wang et al., 2013; Zhang et al., 2018). Accordingly, there are 277 and 255 compound preparations containing Angelicae Sinensis Radix or Astragali Radix, respectively, which have been mentioned by the National Medical Products Administration of China (https://www.nmpa.gov.cn/datasearch/search-result.html). The number of compound preparations that were simultaneously added with Angelicae Sinensis Radix and Astragali Radix was up to 39 (Supplementary Table S4). Thus, the developed specific SNP primers and the multiplex PCR assay will have a wide application in the authentication of Angelicae Sinensis Radix or/and Astragali Radix of the compound preparations.

Regarding the advantages of the multiplex PCR assay, such as being simple, effective, economical, time-saving, and performed without any complicated bioinformatics analysis like DNA metabarcoding and shotgun genomics, it also has some limitations: 1) this method could not be used for authenticating adulterants mixed in authentic crude drugs. It is necessary to further design species-specific primers for all common adulterants, which allow identifying both genuine crude drugs and adulterants within CPM simultaneously. 2) The success of the multiplex PCR assay is closely related to the degree of DNA degradation and the specificity of primers (Wang et al., 2017). We have to fully understand the processing process for producing various kinds of CPM, which will be helpful in obtaining high-quality genomic DNA as much as possible. Likewise, we should also estimate the genetic relationship among the original species of the biological ingredients within CPMs so as to screen high-resolution targeted gene fragments and design highly species-specific primers and ensure the success rate of identification. 3) The quality control of CPM not only requires quantitative evaluation but also qualitative evaluation. In order to determine whether the manufacturer really provides the biological ingredients according to the dosage of the CPM formula, the molecular quantitative method needs to be further developed in the future. Previous studies have reported that the amount of some remnant DNA fragments (DNA copy numbers) could reflect the number of crude drugs required for animal-derived CPM (Ye et al., 2018). Therefore, to simultaneously realize qualitative and quantitative monitoring of biological components within CPM, the fluorescence quantitative PCR and ddPCR assay based on the changes in DNA copy number might be an alternative choice.

## 5 Conclusion

In this study, we first designed two highly species-specific SNP primers and developed a multiplex PCR assay for simultaneous authentication of Angelicae Sinensis Radix and Astragali Radix within the Danggui Buxue pill. The multiplex PCR assay we developed was efficient, sensitive, time-saving, and economical, which could not only be applied to supervise the authenticity of the biological ingredients within the Danggui Buxue pill but also provide a new methodological reference to the qualitative quality control of other CPM.

## Data Availability

The original contributions presented in the study are included in the article/Supplementary Material; further inquiries can be directed to the corresponding authors.
