# Supplementary figures and images for: A specific SNP-based multiplex PCR assay for the simultaneous identification of two biological ingredients for the Chinese patent medicine, Danggui Buxue pill
Source: Front Pharmacol. 2023 May 11;14:1098598. doi: 10.3389/fphar.2023.1098598 (PMC10213767; doi:10.3389/fphar.2023.1098598)

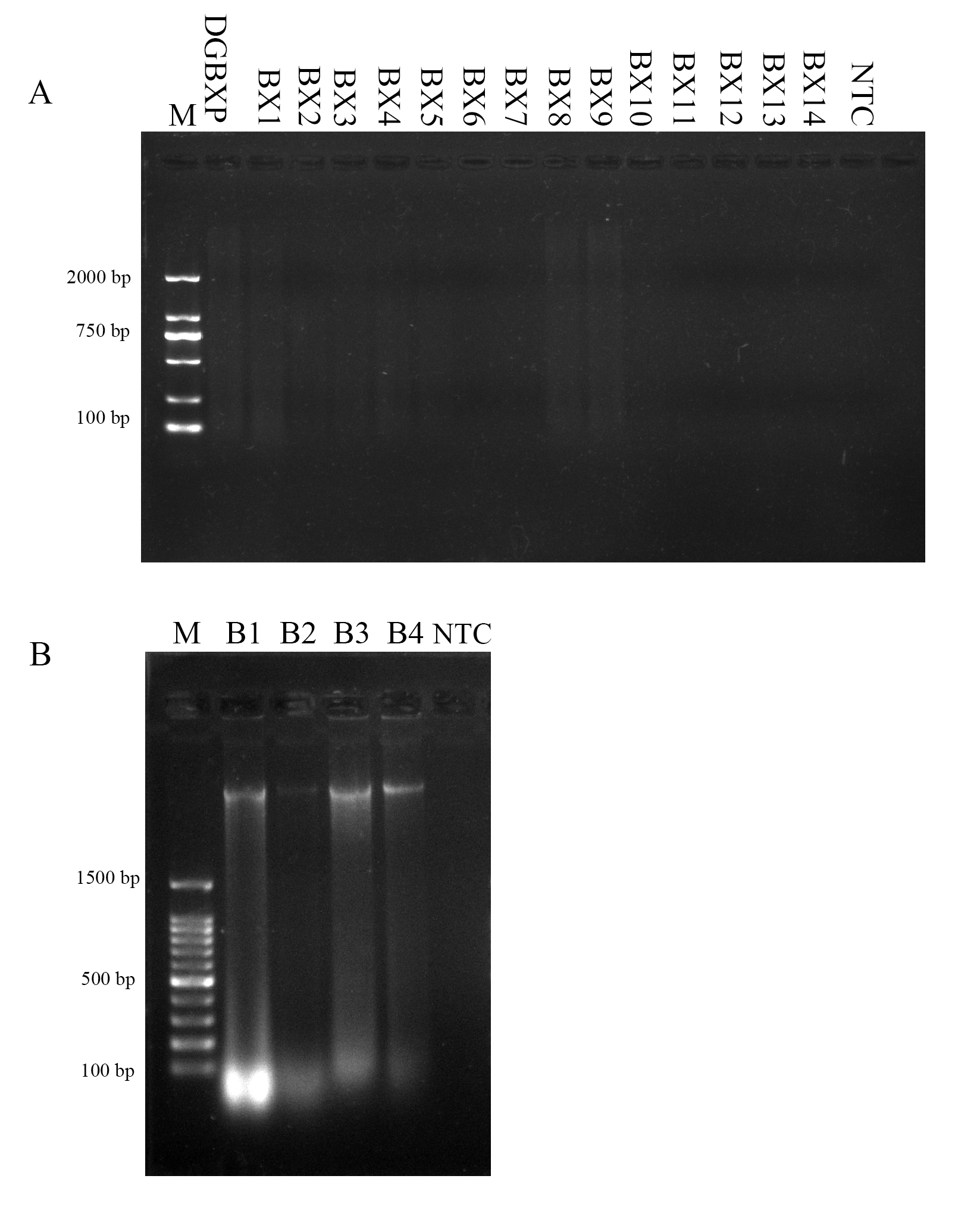

Supplement: Supplementary file 4 [file Image3.TIF]

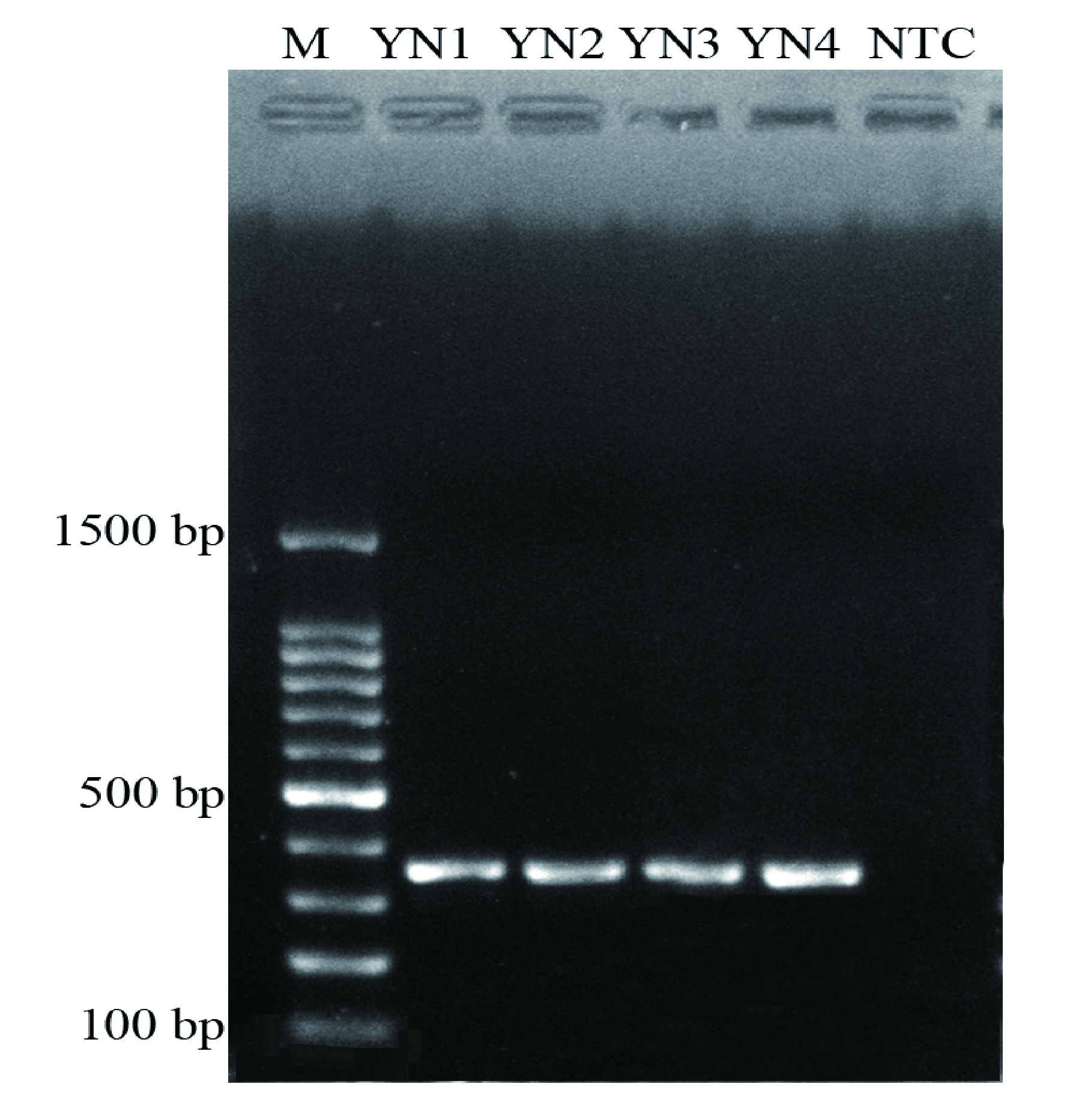

Supplement: Supplementary file 5 [file Image4.TIF]

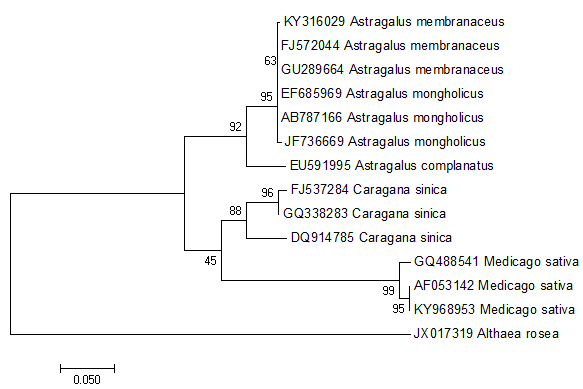

Supplement: Supplementary file 6 [file Image2.TIF]

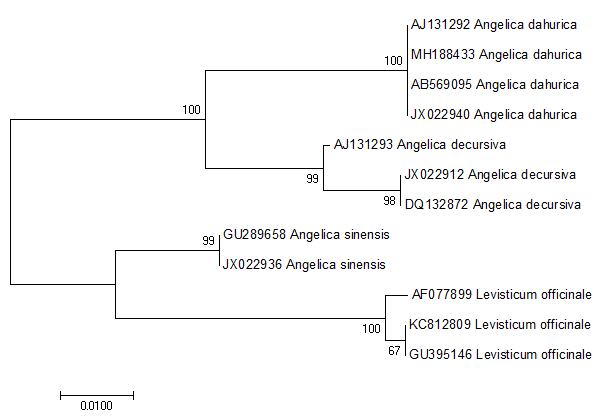

Supplement: Supplementary file 7 [file Image1.TIF]

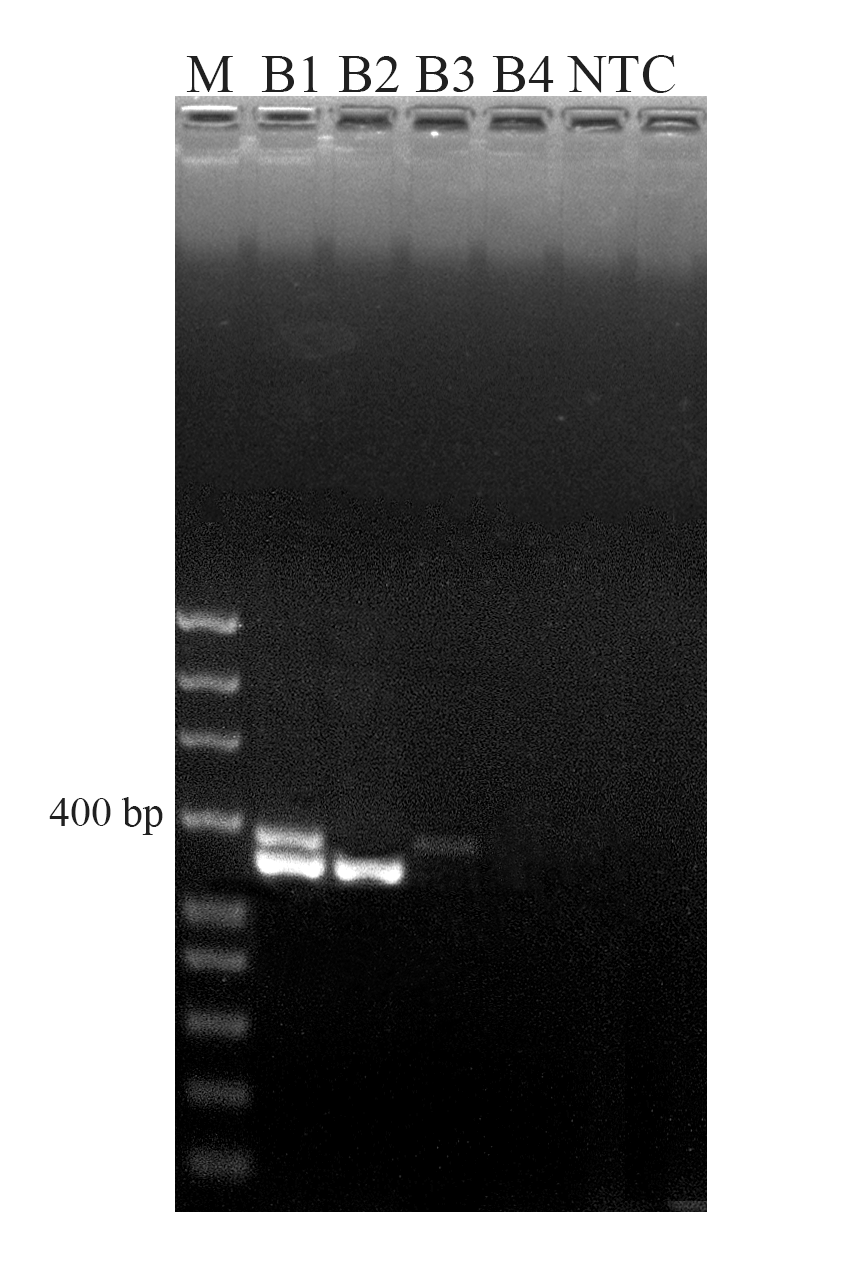

Supplement: Supplementary file 9 [file Image5.TIF]
